# Public engagement lectures targeting prospective medical students: an opportunity for dermatology

**DOI:** 10.1111/ced.13765

**Published:** 2018-10-02

**Authors:** S. Muthiah, K. Wu, N. Rajan

**Affiliations:** ^1^ Department of Dermatology Royal Victoria Infirmary Newcastle upon Tyne UK; ^2^ Department of Dermatology The York Hospital York UK; ^3^ Institute of Genetic Medicine University of Newcastle upon Tyne Newcastle upon Tyne NE1 3BZ UK

Motivation to enter a career in medicine can be underpinned by a variety of factors, including an interest in science, altruistic reasons, and prior personal experiences with doctors and patients.^1^ Early exposure to Dermatology may not capture the clinical variety the specialty offers, and may negatively influence career selection.^2^ Missing from the portrayal of dermatology in the media are the diverse roles that dermatologists play in crosscutting interactions with Paediatrics, Genetics, Epidemiology, Immunology and Oncology. We report our experience of a public engagement initiative tailored to prospective medical students, where we aimed to showcase the diversity offered by Dermatology.

We delivered an interactive lecture to teenagers as part of an annual six‐part lecture series entitled ‘Mini Medical School’. Interaction was facilitated by using live, app‐based, audience polling, which is a method to engage smartphone users that allowed us to obtain anonymized survey data (70% of 340 attendees logged in and took part this year), with results immediately shared with the audience. Audience age ranged from 13 to 18 years (73% female). At the outset, polling indicated that 36% (response rate: 70% of audience) would consider Dermatology as a career choice. We then proceeded to give overviews of common skin disease, UV exposure, ageing, skin cancer and genetics. We focused on ultraviolet (UV) exposure, and interestingly this revealed that 6% of the audience had used sunbeds. Building on this shared result with the audience, we taught about sun protection using methods that involved audience participation. For example, we demonstrated how sunscreens work, by using UV ink to write on the skin of a volunteer and displayed this with a Wood's lamp. This message was then blocked using a sample of sunscreen, which each attendee received. We then trained the audience to recognize features of melanoma, before evaluating their understanding through their ability to correctly identify images as malignant or benign; students were correct in up to 97% of cases based on responses. A combination of didactic and interactive messages was favourably received over a 2‐hour period, at the end of which the same question about Dermatology as a career choice received a 70% response (response rate: 52% of audience). Caveats to our data include sampling from a single centre, and the time‐frame observed precluding long‐term follow‐up.

There are many points in the course of medicine where career choices are shaped, and perhaps we can do more as educators in Dermatology to profile the diversity in our field. For adolescents, early exposure to healthcare can contribute to an increased interest in medicine as a career.^3^ It is therefore of interest to dermatologists to positively engage potential doctors early on in their education. The visual nature of Dermatology lends itself well to audience engagement and participation in interactive lectures. Few people enjoy putting up their hands in a large lecture to speak, but in an era of smartphone‐armed teenagers, clicking within an app (or swiping left) is an increasingly acceptable means to respond. We share our positive experience to encourage the dialogue of how influencing perceptions of Dermatology may evolve to the advantage of our field.
